# The Regulation and Function of Histone Crotonylation

**DOI:** 10.3389/fcell.2021.624914

**Published:** 2021-04-06

**Authors:** Angeliki Ntorla, Joseph Robert Burgoyne

**Affiliations:** The Rayne Institute, School of Cardiovascular Medicine and Sciences, The British Heart Foundation Centre of Research Excellence, King’s College London, St. Thomas’ Hospital, London, United Kingdom

**Keywords:** crotonylation, histone post-translational modification, epigenetics, chromatin, gene regulation

## Abstract

Histone crotonylation is a newly identified epigenetic modification that has a pronounced ability to regulate gene expression. It belongs to an expanding group of short chain lysine acylations that also includes the extensively studied mark histone acetylation. Emerging evidence suggests that histone crotonylation is functionally distinct from histone acetylation and that competition for sites of modification, which reflects the cellular metabolic status, could be an important epigenetic mechanism that regulates diverse processes. Here, we discuss the enzymatic and metabolic regulation of histone crotonylation, the “reader” proteins that selectively recognise this modification and translate it into diverse functional outcomes within the cell, as well as the identified physiological roles of histone crotonylation, which range from signal-dependent gene activation to spermatogenesis and tissue injury.

## Introduction

Histone post-translational modifications (PTMs) constitute a major epigenetic mechanism for the control of gene expression. Histone marks have been detected on various residues, located either within the histone globular domain or along the tail, where they can affect the condensation, packaging, or binding of proteins to chromatin, which intricately regulate processes from gene expression to genomic stability. Due to this functional importance, aberrant patterns of histone PTMs have been implicated in various diseases including cancer and cardiovascular disease (Bannister and Kouzarides, 2011; Abi Khalil, 2014).

Owing to the advancement of high-sensitivity mass spectrometry, that has emerged as the gold standard technique for the identification of novel protein modifications, this has greatly expanded the catalogue of known histone PTMs. This includes the identification of a group of “short chain Lys acylations” that include Lys butyrylation, propionylation (Chen et al., 2007), formylation (Jiang et al., 2007), succinylation, malonylation (Xie et al., 2012), 2-hydroxyisobutyrylation (Dai et al., 2014), b-hydroxybutyrylation (Xie et al., 2016), glutarylation (Tan M. et al., 2014), benzoylation (Huang et al., 2018) and crotonylation (Kcr) (Tan et al., 2011). These modifications are similar to the archetypal Lys acetylation (Kac), but differ in hydrocarbon chain length, hydrophobicity or charge (Table 1). Mounting evidence suggests that these new histone marks can affect gene regulation and are functionally distinguishable from the commonly studied histone Kac, adding another level of complexity to chromatin biology (Sabari et al., 2017).

**TABLE 1:** Summary of histone Lys acylations.

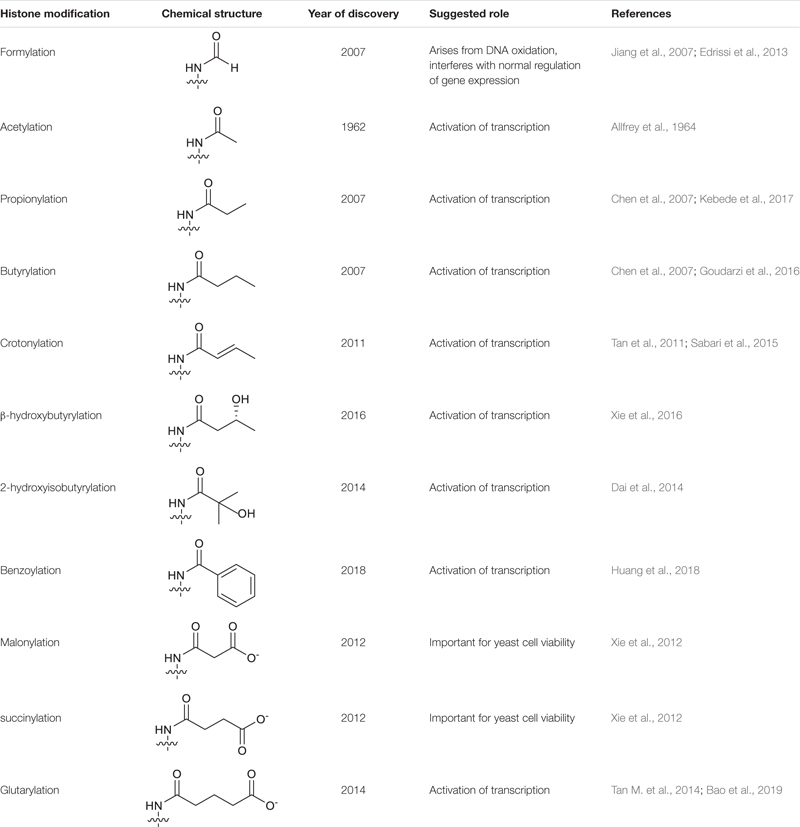

Histone Kcr was first identified in 2011 where it was found to be mainly associated with active chromatin (Tan et al., 2011). Since then, there has been growing interest in this modification as it has emerged as a powerful novel epigenetic mark. Like acetylation, crotonylation also occurs on the ε-amino group of Lys residues and modifies histone charge. Also similar to acetylation, the substrate for crotonylation is a donor molecule linked by a thioester to the sulfhydryl group of coenzyme A (CoA), namely crotonyl-CoA. A key question that has emerged following the discovery of Kcr, as with other newly identified acylations, is whether Kcr is functionally redundant from histone Kac or if it has a distinct role in regulating gene function. Here, the discovery and functional characterisation of histone crotonylation is described, as well as recently identified histone Kcr “writers,” the enzymes that catalyse this covalent modification, “erasers,” the enzymes that remove this modification and “readers,” the effector proteins that bind to histones in a crotonylation-dependent manner. In addition to the enzymatic regulation of histone crotonylation, the impact of cellular metabolism on this epigenetic process are also discussed. Finally, recent advances into the role of histone Kcr in health and disease are described.

## The Discovery of Histone Lys Crotonylation

When histone Kcr was first identified, it was found to be evolutionary conserved from yeast to human, occurring broadly in all core histones (H2A, H2B, H3, and H4), as well as linker histone H1 and marked active promoters and potential enhancers (Tan et al., 2011). Similar to Kac, Kcr also occurs on the ε-amino group of the lysine side chain, where it neutralizes the positive charge of this residue. The loss in positive charge on histone Lys residues weakens DNA interaction, thus making chromatin less compact and accessible to DNA-binding factors. In support of a potential *cis*-function of Kcr on chromatin structure, H3K122cr-H4 containing tetrasomes that were subjected to thermal stability assays, were found to be less stable compared to unmodified H3-H4 tetrasomes (Suzuki et al., 2016). Consistent with this, the ability of Kcr to destabilise nucleosome structure has been proposed to be part of a compensatory mechanism during chromatin-to-nucleoprotamine transition, an essential process during spermatogenesis as discussed in the spermatogenesis section below (Montellier et al., 2013). Montellier et al. (2013) showed that incorporation of a histone H2B variant, TH2B, is essential for the final transformation of dissociating nucleosomes into protamine packed structures. In the absence of TH2B, cells compensate by upregulating H2B and programming nucleosome instability to reach that of wild type cells through targeted histone modifications, including crotonylation of H3K122 and H4K77. This in turn allows the histone replacement to take place. Furthermore, modified histone lysine residues can mediate trans-effects through recruitment of effector proteins containing specific reader modules. This is particularly important for histone Kcr, where the crotonyl group is a four-carbon chain containing a C–C π bond that results in a rigid planar conformation, which is unique among histone acylations. The extended hydrocarbon chain of the crotonyl group increases the hydrophobicity and bulk of the Lys residue compared to acetylation (Sabari et al., 2017). These differences in the biophysical properties of the crotonyl group provide an important mechanism of specificity for reader interaction, as described in detail below.

## Writers and Erasers of Histone Lys Crotonylation

Histone Kcr is dynamically regulated by the opposing enzymatic activities of writers and erasers. To date, no selective enzymes that directly add or remove crotonyl groups from modified lysine residues have been identified, other than previously characterised histone acetyltransferases (HATs) and histone deacetylases (HDACs) (Table 2). Although there is some evidence that histone Lys crotonylation can also occur non-enzymatically, this may be an artefact of *in vitro* conditions in the presence of a high concentration of crotonyl-CoA (Liu S. et al., 2017).

**TABLE 2:** Writers and erasers of histone crotonylation.

Enzyme family	Members
**Writers**	
p300/CBP	p300
	CBP
GNAT	Gcn5
MYST	MOF
	Esa1
**Erasers**	
**Zn^2+^-dependent HDACs**	
Class I HDACs	HDAC1
	HDAC2
	HDAC3
	HDAC8
**NAD^+^-dependent sirtuins**	
Class III HDACs	Sirt1
	Sirt2
	Sirt3

### Writers

In metazoans, HATs are categorised into three major families that are defined by their sequence and structural features: p300/CREB-binding protein (p300/CBP), MYST (MOZ, Ybf2, Sas2, and Tip60) and GCN5-related N-acetyltrasferase (GNAT) family (Roth et al., 2001). Sabari et al. (2015) identified that the well-characterised HAT and transcriptional coactivator p300 also possesses histone crotonyltransferase (HCT) activity *in vitro* and in cells. Consistently, the active site of p300 can accommodate crotonyl-CoA, however histone Kcr activity is much less efficient (by 64-fold) compared to Kac due to steric constraint (Kaczmarska et al., 2017). The crotonyl-CoA is initially positioned in the substrate-binding tunnel adopting an extended conformation which, in contrast to that of acetyl-CoA, is incompatible with lysine binding. Kaczmarska et al. (2017) proposed that engagement of the histone lysine substrate displaces the crotonyl group from the acceptor lysine tunnel into a “back hydrophobic pocket” within the active site in order to enable an orientation suitable for acyl-chain transfer. Although histone Kcr has been detected in various eukaryotes including yeast, no p300/CBP homolog exists in this organism, which suggests that other enzymes responsible for histone crotonylation may exist. Indeed, after the discovery of p300, the MYST family members, human MOF and its yeast homolog Esa1, were also reported to exhibit HCT activity. While Esa1 was found to be responsible for bulk histone crotonylation in budding yeast, in mammalian cells p300 and CBP are the major HCTs (Liu X. et al., 2017). This observation is in contrast to the poor crotonyltransferase activity of p300 identified *in vitro* and implies that other cellular factors, such as potential p300 partners, are required for its enhanced activity towards crotonyl-CoA in cells. Of note, very weak HCT activities were observed for recombinant MOF or Esa1 *in vitro*, suggesting that both proteins are also likely to function in cells as part of a protein complex, or their activity may be regulated by other modifications (Simithy et al., 2017). This is consistent with recent evidence that Esa1 together with the other main HAT in budding yeast, Gcn5, exhibit HCT activity *in vitro* and *in vivo* as part of the ADA and Piccolo NuA4 complexes, respectively (Gcn5-Ada2-Ada3 and Esa1-Yng2-Epl1). Mapping the sites of modification using mass spectrometry has revealed that Gcn5 catalyses crotonylation at Lys residues 9, 14, 18, 23, and 27 of histone H3, while Esa1 crotonylates Lys residues 5, 8, 12, and 16 in histone H4. Notably, the histone residues targeted for crotonylation by Gcn5 and Esa1 are the same sites that these enzymes acetylate (Kollenstart et al., 2019).

### Erasers

Histone deacetylases can be classified into four classes according to sequence similarity: class I, class II, and class IV HDACs that are Zn^+^-dependent, while class III HDACs, also known as sirtuins, are NAD^+^-dependent (De Ruijter et al., 2003). Class I HDAC3 was the first enzyme reported to exhibit histone decrotonylase (HDCR) activity *in vitro*. By profiling HDAC activities using a library of fluorogenic substrates, only HDAC3 in complex with nuclear corepressor 1 (NCoR1) demonstrated a measurable HDCR activity, even though this was diminished compared to its deacetylase activity (Madsen and Olsen, 2012). Recently, further studies have demonstrated that in addition to HDAC3, all other class I members, HDAC1, 2, and 8, exhibit robust HDCR activities *in vitro*, while class II and class IV HDACs have failed to display any HDCR activity (Wei et al., 2017; Fellows et al., 2018; Kelly et al., 2018). In addition, sirtuins can also exhibit HDCR activity. A comprehensive analysis of the activity of the seven mammalian sirtuins using H3 peptides carrying diverse acyl groups on Lys 9, revealed HDCR activity for Sirt1 and Sirt2 (Feldman et al., 2013). In a successive study, Sirt3 was also found to have HDCR activity *in vitro*, with its knock-down leading to increased histone Kcr that was associated with enhanced gene expression (Bao et al., 2014). In addition, the knock-down of HDAC1, HDAC2, or HDAC3 in HeLa cells or attenuation of their activity using the HDAC-specific inhibitor trichostatin A, resulted in elevated histone crotonylation and acetylation. Moreover, this effect was further enhanced with the simultaneous knock-down of HDAC1/2/3. However, selective knockdown of either SIRT1 or SIRT3 did not significantly impact on overall histone crotonylation, neither did the concurrent knockdown of SIRT1/3/5. These findings suggest the class I HDACs are likely to be the major HDCRs in mammalian cells (Wei et al., 2017). This is also consistent with genetic deletion of HDAC1/2 in embryonic stem (ES) cells, which resulted in increased global levels of histone crotonylation and caused an 85% reduction in total HDCR activity. Also, loss of HDAC1/2 led to enrichment of H3K18cr around transcription start sites, which largely overlapped with H3K18ac and correlated with gene activity (Kelly et al., 2018).

### Other Regulators

**FIGURE 1:**
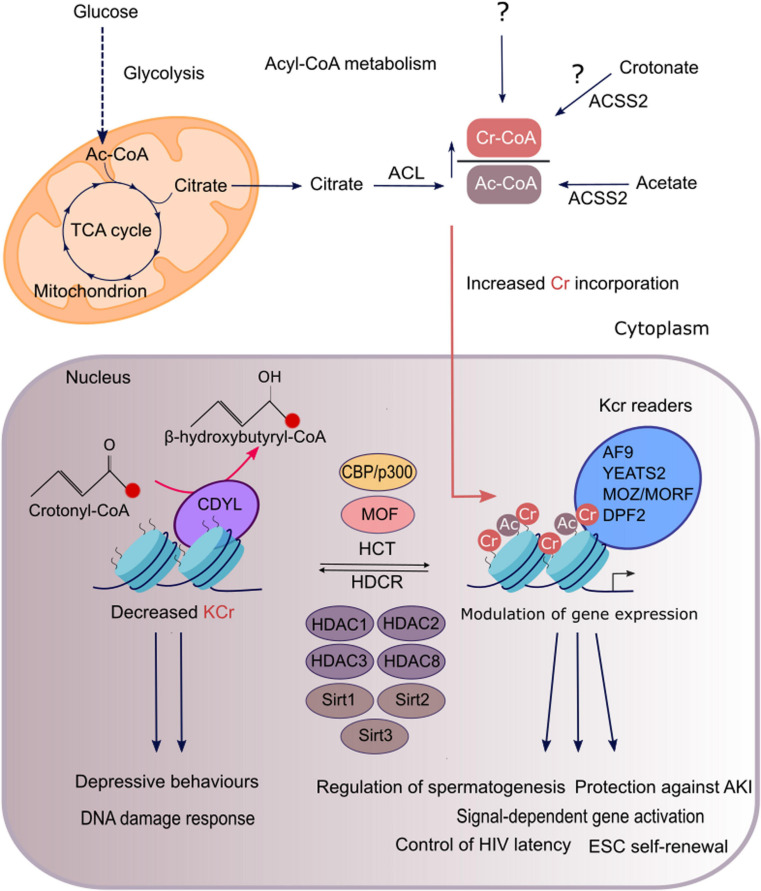
Regulation and functions of histone crotonylation in mammalian cells. In genomic regions that are regulated by both histone crotonylation and acetylation, the degree of each modification is determined by the relative intracellular concentration of acetyl-CoA and crotonyl-CoA, which are produced through cellular metabolic pathways shown here. Acetyl-CoA is mainly synthesised from mitochondrial citrate, derived from glucose oxidation, by the enzyme ACL. Short-chain fatty acids, acetate and crotonate, can be converted to their cognate acyl-CoAs, that is mediated by ACSS2 at least for acetyl-CoA. Crotonyl-CoA is also generated as a by-product of fatty acid and amino acid metabolism. However, it remains unknown whether crotonyl-CoA generated through these pathways or an alternate route supplies the nuclear pool of crotonyl-CoA, which acts as a substrate for histone crotonylation (as indicated by the question mark). ACL and ACSS2 reactions can take place in both the cytoplasm and the nucleus. For simplicity only the cytosolic reactions are depicted. A high ratio of crotonyl-CoA to acetyl-CoA will favour the incorporation of crotonyl moieties into the chromatin by acyltransferases. The HATs p300/CBP and MOF have been characterised as crotonyltransferases (HCTs) while class I HDACs are the major HDCRs in mammalian cells. Some sirtuins (SIRT1,2,3) also exhibit HDCR activity. In addition, the chromodomain protein CDYL is a negative regulator of histone crotonylation, as it converts crotonyl-CoA to β-hydroxybutyryl-CoA, thus limiting the substrate available for histone crotonylation. Histone Kcr is selectively recognised by reader proteins which include YEATS2, AF9, MOZ, MORF, and DPF2 that can subsequently translate it into diverse functional outcomes. Histone crotonylation exerts diverse functions such as in gene activation, spermatogenesis, kidney injury, depression, DNA damage response, HIV latency, and maintenance of stem-cell renewal.

The chromodomain Y-like transcription co-repressor (CDYL) is a chromatin reader protein that constitutes part of a repressive chromatin complex needed for the transmission and restoration of repressive histone marks, which preserves the epigenetic landscape, important for maintaining cell identity (Liu Y. et al., 2017). In addition to its reader function, CDYL also regulates histone crotonylation as it has crotonyl-CoA hydratase activity (Figure 1). This activity has been suggested to be intrinsically linked to the transcription repressive function of the protein (Liu S. et al., 2017). CDYL contains an N-terminal chromodomain and a C-terminal enoyl-CoA hydratase/isomerase homology domain (also known as CoA pocket or CoAP) (Caron et al., 2003). Consistent with the identified crotonyl-CoA hydratase activity of CDYL, the CoAP domain of the protein has been shown to be able to bind CoA while both the chromodomain and the CoAP domain are required for its negative regulation of histone Kcr, suggesting that CDYL mediated hydratation of crotonyl-CoA occurs when the protein is bound to chromatin (Caron et al., 2003; Liu S. et al., 2017). Of note, Caron et al. (2003) showed that in addition to CoA, the CoAP domain can also bind HDACs and that HDAC1/2 binding abolishes the ability of CDYL to bind CoA. These findings support the notion that transcription repression by CDYL is due to its reader function could be separate from its activity as a metabolic enzyme. Although little is known about the potential intrinsic transcriptional repressive activity of CDYL, its crotonyl-CoA hydratase activity has been exploited in studies to investigate the functional role of histone crotonylation (Liu S. et al., 2017; Liu et al., 2019). However, due to the reader activity of CDYL and its ability to repress transcription, which are independent of histone crotonylation, it is difficult to discern the functional impact of this protein that can be truly attributed to a change in this epigenetic mark (Zhang et al., 2011; Mulligan et al., 2019).

## Readers of Histone Lys Crotonylation

**FIGURE 2:**
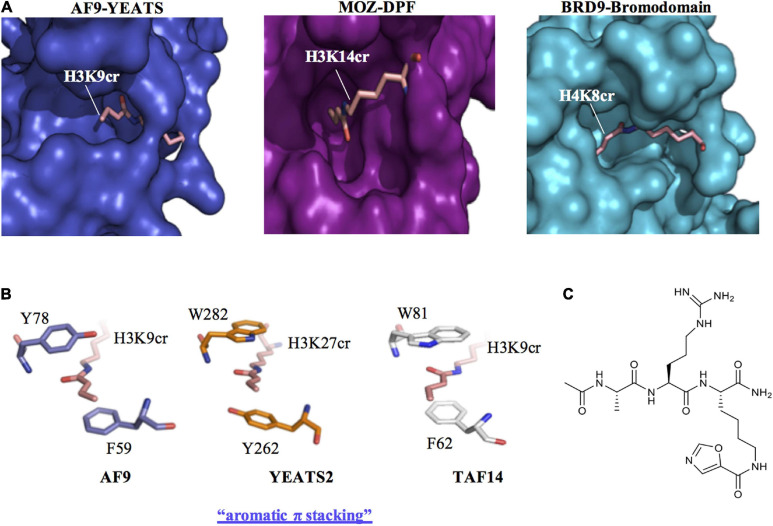
Recognition of histone crotonylation by acyllysine readers. **(A)** Comparison of the binding pockets of AF9-YEATS [Protein Data Bank Identifier (PDB ID): 5HJB], MOZ-DPF (PDB ID: 5B76) and BRD9-bromodomain (PDB ID: 4YYH) reader proteins in complex with histone peptides that contain crotonyl-lysine (H3K9cr, H3K14cr, and H4K8cr, respectively). **(B)** Crystal structures of the YEATS domains of AF9 (PDB ID: 5HJB), YEATS2 (PDB ID: 5IQL), and TAF14 (PDB ID: 5IOK) in complex with crotonylated histone peptides reveal a conserved molecular mechanism for crotonyllysine recognition among the YEATS family members. The sandwiching of the planar crotonylamide group between two aromatic residues, which is also known as aromatic π stacking, explains the preference of the YEATS proteins for Kcr. **(C)** Structure of the YEATS domain selective inhibitor XL-13m.

Early recognition of the physiological relevance of histone crotonylation prompted studies into the identification of candidate chromatin-associated proteins that are able to “read” this mark. These efforts focused on classical members of the three major families of histone Kac readers, which were examined for their ability to recognise the unique structure of the crotonyl group conjugated to histone lysine residues. These include bromodomains, YEATS (Yaf9, ENL, AF9, Taf14, and Sas5) and double plant homeodomain finger (DPF) domains proteins (Sabari et al., 2017; Figure 2). By analysing the crystal structure of the human AF9 YEATS domain in complex with H3K9ac, Li et al. (2016) speculated that the YEATS domain could preferentially accommodate longer and bulkier acyl groups, due to an open space within the binding pocket (Figure 2A). This was substantiated in subsequent studies where the YEATS domain was found to have a preference for binding acyl chains longer than acetyl, with the strongest affinity for Kcr (Li et al., 2016). Notably, YEATS domains have a preference for Kcr binding by ∼2–7-fold compared to Kac (Zhao et al., 2017). By using a peptide array and isothermal titration calorimetry it was revealed that AF9 YEATS recognizes histone H3 crotonylation at K9, K18, and K27 with highest affinity for H3K9cr. In addition, the YEATS domain of yeast Taf14 was found to have a similar preference for binding to histone H3 crotonylation as AF9 YEATS. In contrast, the YEATS domain of YEATS2 is selective for histone H3K27cr (Li et al., 2017). The preferential binding of the YEATS domain to sites of Kcr is a result of a unique “aromatic-π-aromatic” stacking (also called “π-π-π” stacking) where the planar crotonylamide group is sandwiched by two aromatic residues, in addition to hydrophobic interactions introduced by a hydrocarbon extension. This aromatic π stacking mechanism for Kcr recognition is consistently observed in the crystal structures of AF9, YEATS2, and Taf14 in complex with Kcr (Figure 2B; Andrews et al., 2016; Li et al., 2016; Zhang et al., 2016; Zhao et al., 2016). In contrast, bromodomains adopt a side-open pocket that generates a spatial restraint that limits their interaction with Kcr. The second bromodomain of TAF1 and the BRD9 bromodomain are among the few examples of this type of reader module that can bind Kcr, albeit with a limited affinity compared to its Kac cognate (Figure 2A; Flynn et al., 2015). Therefore, the YEATS domain proteins represent the first class of selective Kcr readers.

Based on the resolved structure of the YEATS-Kcr complex, Li et al. (2018) developed the first class of YEATS domain selective inhibitors. Among a series of peptide-based molecular probes, one was optimised to selectively target the ENL YEATS domain (XL-13m), which has been previously implicated in the regulation of the oncogenic transcriptional program in acute leukaemia (Figure 2C). The peptide XL-13m was found to associate with endogenous ENL, disrupting the recruitment of ENL onto chromatin, and synergizing with BET and histone methyltransferase DOT1L inhibitors, leading to enhanced downregulation of a set of oncogenes in MLL-rearranged acute leukaemia (Li et al., 2018). Given the preference of YEATS domain proteins for Kcr over Kac, inhibitors developed in this study can be exploited to further investigate the physiological and pathological role of histone Kcr.

The DPF domains of the MYST family member monocytic leukemic zinc-finger (MOZ, also known as KAT6A) and DPF2 also exhibit diverse reader activity with the highest binding affinity for Kcr (Xiong et al., 2016). This was revealed using isothermal titration calorimetry, where DPF domains were found to have a 4–8-fold enhanced affinity for Kcr compared to Kac, while in affinity pull-downs MOZ and DPF2 were found to have specificity for H3K14cr. Although the DPF domains display similar Kcr selectivity as YEATS, the underlying mechanism for this preference in distinct. In the crystal structure of the DPF domain of MOZ in complex with H3K14cr, it was revealed that the interaction was through an intimate hydrophobic pocket that lacked aromatic sandwiching residues, which are characteristic of the YEATS domains (Figure 2A). The importance of this mechanism of Kcr interaction with DPF domains was revealed using ChIP-qPCR and immunofluorescence, where MOZ colocalised with sites of H3K14cr in cells in a manner dependent on its DPF domain (Xiong et al., 2016). In a more recent study, the DPF domain of the HAT complex MOZ-related factor (MORF), was also shown to preferentially bind H3K14cr over H3K14ac. Moreover, binding of the DPF domain to H3K14cr enhanced the catalytic activity of MORF towards another acetylation site (H3K23ac), highlighting the interrelated nature of different PTMs (Klein et al., 2019).

## Metabolic Regulation of Histone Lys Crotonylation

The donor for histone crotonylation, crotonyl-CoA, is an important intermediate involved in several cellular metabolic pathways including fatty acid and amino acid metabolism. The synthesis of crotonyl-CoA can also occur in the mitochondria or the cytoplasm. However, the metabolic sources and mechanisms responsible for generating the nuclear pool of crotonyl-CoA that fuels histone crotonylation remain unknown. Mounting evidence suggests that histone acylations are directly sensitive to changes in the concentrations of their corresponding acyl-CoA metabolites, and therefore can act as indicators of the cellular metabolic state (Simithy et al., 2017).

Sabari et al. (2015) provided initial evidence that histone crotonylation can be regulated metabolically through pathways that influence the cellular concentrations of crotonyl-CoA (Figure 1). Here the addition of the short chain fatty acid (SCFA) crotonate to HeLa S3 cells was found to dramatically increase both the cellular concentration of crotonyl-CoA and H3K18cr in a dose-dependent manner. Crotonate, like other SCFAs, is mainly produced by the gut microbiota during the fermentation of partially and nondigestible carbohydrates (Tan J. et al., 2014). Circulating SCFAs (acetate, crotonate, butyrate, and propionate) can be taken up by tissues and converted into their cognate short-chain acyl-CoAs, the direct donors of histone Lys acylations. This is consistent with depletion of the gut microbiota in mice with antibiotics, which led to a reduction in luminal and serum SCFAs with a concomitant decrease in histone crotonylation (Fellows et al., 2018). Nevertheless, endogenous sources of crotonate are unclear. The mechanism for the action of crotonate in HeLa cells was explained by its cellular uptake, leading to its conversion to crotonyl-CoA by the metabolic enzyme acyl-CoA synthetase 2 (ACSS2 or AceCS1), which is also known to generate acetyl-CoA from acetate. Intriguingly, the knock-down of ACSS2 led to a reduction in basal histone Kcr, suggesting that crotonate might be a physiologically relevant source of crotonyl-CoA. However, the ability of ACSS2 to synthesize crotonyl-CoA from crotonate has not been directly demonstrated with *in vitro* assays. Hence, the possibility of an indirect reduction of histone crotonylation upon ACSS2 knockdown cannot be ruled out. Indeed, ACSS2 knock-down leads to a reduction of the cellular pool of acetyl-CoA, which is used for fatty acid synthesis and hence beta oxidation of fatty acids, both required for crotonyl-CoA production. In fact, an alternative potential source of crotonyl-CoA in metazoans is through metabolic pathways that include fatty acid β-oxidation or the metabolism of the essential amino acids lysine or tryptophan. The sequential breakdown of fatty acid molecules by mitochondrial β-oxidation to form acetyl-CoA, leads to generation of a crotonyl-CoA intermediate upon oxidation of butyryl-CoA, catalysed by acyl-CoA dehydrogenase. In addition, degradation of the essential amino acids lysine or tryptophan within the mitochondria also generates a crotonyl-CoA intermediate upon oxidative decarboxylation of glutaryl-CoA, catalysed by glutaryl-CoA dehydrogenase. Once crotonyl-CoA is formed by either the fatty acid β-oxidation or amino acid degradation pathways, it undergoes hydration to 3-hydroxybutyryl-CoA that is catalysed by enoyl-CoA hydratase. However, some crotonyl-CoA may escape degradation and instead leak from the mitochondria where it can then contribute to histone crotonylation. This would be consistent with evidence that histone crotonylation is mediated by the fatty acid β-oxidation pathway in yeast (Gowans et al., 2019).

Based on the dual enzymatic activity of p300, it was speculated that crotonyl-CoA and acetyl-CoA could compete to influence the type of acylation. So far very few studies have attempted to measure the relative intracellular concentrations of acetyl-CoA and crotonyl-CoA. In such an effort, crotonyl-CoA was found to be ∼1,000-fold less abundant compared to acetyl-CoA in various cell types (HeLa cells and myogenic cells) (Sabari et al., 2015; Simithy et al., 2017). However, when the cellular pool of citrate-derived acetyl-CoA is depleted by knocking down ATP citrate lyase, this not only decreases acetylation of H3K18 as expected (Wellen et al., 2009), but also increases p300-catalysed crotonylation (Sabari et al., 2015). In addition, when pyruvate dehydrogenase is knocked down, an enzyme recently found to produce a nuclear pool of acetyl-CoA from pyruvate needed for histone acetylation (Sutendra et al., 2014), this also promoted H3K18cr. Together these findings support a model where crotonyl-CoA can compete with acetyl-CoA for p300’s acyltransferase activity.

## Functions of Histone Lys Crotonylation

### Gene Regulation

Although histone crotonylation was originally associated with active chromatin, it was not until 2015 when Sabari et al. (2015) functionally characterised this modification, that it was confirmed to be a positive regulator of transcription. By utilizing a cell-free transcription assay, in the presence of either crotonyl-CoA or acetyl-CoA, p300 catalysed histone crotonylation was found to stimulate transcription that was more potent than acetylation. In addition, in macrophages stimulated with lipopolysaccharides (LPS), inflammatory gene expression was enhanced by p300, and was further potentiated by Kcr. Initially, LPS stimulation resulted in elevated Kac and Kcr at the promoters of inflammatory genes. However an increase in the concentration of intracellular crotonyl-CoA (by crotonate pre-treatment) prior to the endotoxin (LPS) treatment, promoted site-specific H3K18cr of the inflammatory genes in a dose-dependent manner. This enrichment of crotonylation correlated with a higher expression of target genes that was also associated with a concomitant decrease in H3K18ac (Sabari et al., 2015). In a follow-up study, the YEATS-domain protein AF9 was found to play an important role in driving active transcription of LPS-stimulated genes that was mediated by H3K18cr. Here, knockdown of AF9 significantly limited the Kcr-dependent response, but this was not fully attenuated. This suggests that other reader proteins could be involved or that Kcr exerts its effects through a reader independent cis-mechanism, which is dependent on nucleosome stability or inter-nucleosomal interactions (Li et al., 2016). Consistent with these findings, a novel CBP/p300 mutant with deficient HAT but competent HCT activity is able to compensate for loss of endogenous CBP/p300 and promote TGFβ-induced transcription (Liu X. et al., 2017).

Although histone Kcr is mainly associated with active transcription, recent studies have also implicated a potential role for this mark in the negative regulation of gene expression (Gowans et al., 2019; Kollenstart et al., 2019). In one of these studies, Gowans et al. (2019) took advantage of the highly synchronised yeast metabolic cycle (YMC) and demonstrated that histone Kcr and Kac show temporarily distinct patterns, which also correlate with diverse gene expression. While both modifications were found to dynamically fluctuate across the YMC yet each one peaks at discrete time points. Interestingly, the highest levels of H3K9cr were observed at the time point of the YMC when H3K9ac was diminished and energy availability became limited. Another characteristic of this phase is the decreased expression of pro-growth genes. Here, by generating a mutant form of Taf14 YEATS protein that lacks H3K9cr reader activity, the authors demonstrated that this results in upregulation of these pro-growth genes. On the other hand, exogenous addition of sodium crotonate resulted in elevated histone crotonylation that was concomitant with constitutive repression of pro-growth genes, and disturbed YMC oscillations. Collectively these results suggest an important role of Taf14-H3K9cr interaction for the normal function of the YMC, potentially through the repression of pro-growth gene expression (Gowans et al., 2019). However, it is still unclear whether the latter is mediated by enrichment of Kcr or binding of Taf14 or other selective readers of Kcr on regulatory elements of downregulated genes. Currently, there is no structural evidence supporting chromatin compaction as a result of histone Kcr. Therefore, select Kcr readers that are involved in transcriptional repression or an inhibitory effect of this modification on binding of transcription factors, could also explain this disparate action of histone Kcr as a suppresser of gene expression. This would also be consistent with the functions already attributed to the similar widely studied mark histone methylation (Curradi et al., 2002). Therefore, more studies are needed to fully elucidate the role of Kcr in mediating gene transcription.

### Acute Kidney Injury

In acute kidney injury (AKI), Kcr has been found to have a nephroprotective role (Ruiz-Andres et al., 2016). A global increase in kidney histone Kcr was observed in mice with experimental AKI induced either by folic acid or cisplatin treatment. This increase in histone Kcr could also be replicated in cultured epithelial tubular cells, triggered by the proinflammatory cytokine tumour necrosis factor-like weak inducer of apoptosis (TWEAK), a key contributor to kidney injury. In TWEAK-treated murine tubular cells and in kidneys from mice with AKI, an enrichment of Kcr was observed in the promoters of *Pgc-1a* (*Ppargc1a*) and *Sirt3*, two nephroprotective genes whose expression diminishes in AKI. In addition, exogenous crotonate administration resulted in a global increase in Kcr in tubular cells in culture and in healthy kidneys *in vivo*. This increase in Kcr was correlated with elevated PGC-1α and SIRT3 expression and decreased expression of CCL2, which encodes a chemokine known to contribute to kidney inflammation. Importantly, systemic crotonate administration protected mice against experimental AKI and preserved their renal function. The prevention of PGC-1α and SIRT3 downregulation as well as CCL2 upregulation, all downstream effects of crotonate administration, provide a potential mechanism for the beneficial role of Kcr in renal injury. Together, these results suggest that increased histone Kcr is a compensatory protective mechanism in the mouse kidney tissue upon AKI. Also, using crotonate to manipulate *in vivo* histone Kcr may provide a potential therapy for the treatment of kidney damage. However, further studies are needed to fully elucidate the role of histone Kcr during AKI.

### Spermatogenesis

Following its discovery, one of the first functions identified for histone Kcr was in mouse spermatogenesis (Tan et al., 2011). During the meiotic stage of spermatogenesis, a major event that takes place is the transcriptional silencing of the X and Y chromosomes, which is known as meiotic sex chromosome inactivation (Turner, 2007). An important stage in this process is the reactivation of specific sex chromosome-linked genes in post-meiotic round spermatids, especially those that are X-linked. Here, these “escapee” genes specifically gain Kcr marks (Tan et al., 2011). Another important phenomenon during mammalian spermatogenesis is the genome-wide removal of histones and their stepwise replacement first by transition proteins and then by protamines. Although global histone hyperacetylation is known to be associated with histone removal in elongating spermatids, Kcr was also found to be present during this process, suggesting this mark is also likely to play an important role (Montellier et al., 2012). Intriguingly, Kac and Kcr showed distinct genomic distributions, therefore each modification is likely to have a diverse role. This is consistent with CDYL catalysed downregulation of Kcr, which resulted in dysregulated histone replacement in the testis of CDYL transgenic mice compared to wild-type, as well as decreased expression of sex-chromosome-linked escaped genes in postmeiotic round spermatids (Liu S. et al., 2017). Moreover, the Cdyl transgenic mice had reduced sperm count and motility, as well as impaired fertility.

### Depression

A role for histone Kcr has been identified in the medial prefrontal cortex (mPFC), a region of the brain that has been associated with the pathology of major depressive behaviours, using a well-established chronic social defeat stress (CSDS) model (Liu et al., 2019). Histone Kcr was downregulated in the mPFC of susceptible mice that were exposed to CSDS. Here, the negative regulator of histone Kcr, CDYL, was also found to be upregulated. In complimentary experiments, when CDYL was knocked-down in the mouse prelimbic cortex of the brain, this resulted in an increase of histone Kcr and prevented stress-induced depressive behaviours. These studies indicate that CDYL is a key mediator of stress-induced alterations in histone Kcr. To identify genes regulated by CDYL, comparative RNA-seq was performed on brain tissue of mice susceptible to defeat stress as well as from naïve mice overexpressing CDYL. Importantly, among a set of genes that were found to be downregulated in both datasets, *Vgf* was identified, which encodes for a neuropeptide that has been previously reported to be diminished in patients with depression and can mediate anti-depressant responses in mice (Hunsberger et al., 2007; Jiang et al., 2017). Extensive analysis demonstrated that CDYL inhibits VGF expression mainly through its dual effect on promoter histone Kcr and site-specific H3K27me3. Here, the CDYL-VGF axis interrupts structural synaptic plasticity in the mPFC contributing to the behavioural changes observed in susceptible mice.

### HIV Latency

The establishment of a latent human immunodeficiency virus (HIV) reservoir, hidden from the immune system of infected individuals who are under suppressive antiretroviral therapy, hampers the ability to cure HIV. Current research efforts towards an HIV therapy are focused on a strategy aiming to reverse latency and hence reveal the latent viral reservoir so it can then be attacked and cleared by a native or engineered immune response of infected individuals. Histone epigenetic modifications are potential targets for therapy as they can regulate both the formation and maintenance of this latent reservoir (Turner and Margolis, 2017). This includes crotonylation of histone tails on the HIV long terminal repeats (LTR) that can control HIV latency (Jiang et al., 2018). The upregulation of histone crotonylation at the HIV LTR mediated by ACSS2 induction, reactivated latent HIV *in vitro* and *ex vivo*, while ACSS2 inhibition attenuated HIV replication and reactivation mediated by histone Kcr. Therefore, histone crotonylation represents a potential novel therapeutic target to eradicate HIV.

### DNA Damage Response

DNA damage response (DDR) is the collective term that is used to describe the intracellular processes that have evolved to combat endogenous- and exogenous-mediated DNA lesions and ensure DNA repair (Jackson and Bartek, 2009). Notably, sensing of DNA lesions and activation of downstream signalling pathways for their repair are largely governed by histone PTMs (Machour and Ayoub, 2020). A recent study has implicated a role for histone crotonylation in DDR and also describes a hitherto unrecognised role of HDACs in regulating Kcr during DNA damage (Abu-Zhayia et al., 2019). Intriguingly, histone crotonylation was found to be downregulated at sites of DNA damage. Specifically, H3K9Cr was found to exhibit a rapid and transient decrease upon the induction of different types of DNA damage caused by ionizing radiation, etoposide treatment or ultraviolet radiation. This damage-induced reduction in Kcr was dependent on the decrotonylase activity of HDACs, which are known to accumulate at sites of DNA damage. Due to the dual enzymatic activity of HDACs in regulating both Kac and Kcr, the development of a strategy to selectively target either activity is required to elucidate the contribution of each modification to DDR.

### Stem Cell Biology

In mouse ES cells histone Kcr is elevated when compared to differentiated cells (Wei et al., 2017). This enrichment in histone Kcr is required for maintenance of ESC self-renewal. This is consistent with studies in which overexpression of wild-type HDAC1 in ESCs led to a marked downregulation of pluripotency markers with a concomitant upregulation in indicators of differentiation. In addition, these changes were accompanied with a drastic reduction in histone Kcr. Moreover, expression of a mutant form of HDAC1 with intact HDCR but defective HDAC activity triggered similar gene responses, indicating that selective histone decrotonylation can promote ES cell differentiation (Wei et al., 2017).

In recent years, reprogramming of somatic cells to produce induced pluripotent stem cells (iPSCs), that closely resemble ES cells, has provided an attractive source for stem cell-based therapies. In an effort to better characterise chemically induced PSCs, Fu et al. (2018) identified an enhanced role for histone crotonylation during chemical reprogramming. When histone crotonylation was induced by addition of crotonic acid, this activated two-cell stage specific genes, including *Zscan4* and increased telomere sister chromatid exchange, which maintain telomere length and reduce telomeric damage during chemical induction, overall improving induction efficiency.

## Conclusion and Future Perspectives

The repertoire of histone PTMs has greatly expanded in recent years, adding further complexity to the field of chromatin biology (Barnes et al., 2019). This includes, the discovery of a group of short-chain Lys acylations that are structurally similar to Kac and provide a link between cellular metabolism and gene regulation (Sabari et al., 2017). Among these newly identified modifications, histone Kcr is an evolutionary conserved epigenetic mark with a pronounced ability to regulate gene expression (Tan et al., 2011; Sabari et al., 2015). Since its discovery there has been mounting evidence for the functional importance of this modification.

Originally histone Kcr was perceived to fulfil a similar role to Kac, as both share sites of modification, writers and erasers (Zhao et al., 2018). However, the identification of reader modules, such as the YEATS domains proteins, that selectively recognize Kcr indicates that these two modifications are interpreted differentially, thus providing diversity in their functional outcome (Li et al., 2017). In addition, the extent of histone Kcr is also metabolically regulated by the cellular concentration of crotonyl-CoA (Sabari et al., 2015). Therefore, changes in the relative abundance of intracellular concentrations of crotonyl-CoA and acetyl-CoA will influence their respective acylations, mediated by the HAT and co-activator p300. This coupled to difference in reader interaction provides a mechanism for diverse signalling. Furthermore, some genomic regions, such as the “escapee” spermatogenesis genes, are also exclusively marked by Kcr and not Kac (Tan et al., 2011). How histone Kcr can be exclusively marked remains unknown. It is anticipated in future studies, novel proteins that interact with or regulate sites of crotonylation will be identified, including Kcr modifying enzymes or additional Kcr selective readers. This may also include writers or erasers that are specific for histone Kcr, which may explain how some sites can be exclusively marked with this modification.

Despite evidence supporting a unique role of histone Kcr in the regulation of specialised transcriptional programs, one cannot overlook the existing similarities between histone crotonylation and histone acetylation. In fact, ChIP-seq mapping with parallel analysis of histone crotonylation and histone acetylation demonstrated a significant overlap between these two marks at genomic locations in human somatic cells (Tan et al., 2011). This observation also holds true for other recently discovered short-chain histone acylations. For instance, ChIP-seq analyses mapped histone acetylation to similar locations, mostly promoters, with histone propionylation and histone butyrylation in hepatic, and spermatogenic cells (Goudarzi et al., 2016; Kebede et al., 2017). More interestingly, the co-occurrence of these marks at transcription start sites of active genes was positively correlated with gene expression. This suggests that these marks are likely to act in combination to promote a high transcriptional outcome. In spermatogenic cells, a competing nature between histone butyrylation and histone acetylation was reported (Goudarzi et al., 2016). Despite acting as a transcriptional activator, histone butyrylation was found to compete with acetylation to prevent binding of the testis specific BET bromodomain factor, Brdt, an important protein that regulates gene expression and histone-to-protamine transition. In late spermatogenic stages, histone acetylation is important for Brdt-dependent histone removal. Butyrylated histones survive this wave of acetylation-dependent histone removal which is consistent with the inability of Brdt to recognize butyrylation. Although histone crotonylation was not examined in this study, the inability of BET factors to bind histone crotonylation could also explain the identified persistence of histone marks in elongating spermatids (Tan et al., 2011; Flynn et al., 2015). These studies suggest that the rapid turnover between modifications, that in turn allows a dynamic association of reader proteins might be important for diverse transcriptional responses and therefore, these marks are likely to act together to coordinate particular transcriptional programs.

Histone Kcr can regulate diverse physiological functions ranging from gene activation to spermatogenesis (Sabari et al., 2015; Liu S. et al., 2017). Furthermore, it can mediate both protective and adverse functions in the development of different diseases (Ruiz-Andres et al., 2016; Liu et al., 2019). However, only a small number of Kcr sites in human histones have been identified so far (Tan et al., 2011). This is in part due to the lack of commercially available Kcr site-specific antibodies, which has meant much of the research in this field has focused on studying total histone crotonylation. This is likely to limit our understanding of the importance of histone Kcr, as the functional impact of modification at specific sites cannot be readily assessed. Moreover, as the histone code dictates, epigenetic responses are a result of a complex interplay between different PTMs (Bannister and Kouzarides, 2011). Furthermore, as the writers and erasers that regulate histone crotonylation also mediate histone acetylation, it is often difficult to discern the functional role of each modification. Although CDYL has been used to target histone crotonylation, due to its nuclear crotonyl-CoA hydratase activity, it lacks specificity as it also functions as a reader of methylated histone, as well as having other transcriptional repressive activities (Zhang et al., 2011; Liu Y. et al., 2017; Liu et al., 2019). Therefore, there is a need for improved access to tools to study histone Kcr, to allow a greater understanding of how this modification contributes to the regulation of physiological and pathological processes.

The intracellular abundance of crotonyl-CoA is considered relatively low, therefore small fluctuations in crotonyl-CoA concentration will likely have a pronounced impact on crotonyltransferase reactions that mediate histone Kcr (Sabari et al., 2015). Accordingly, the investigation of pathways that fuel the generation of crotonyl-CoA destined for histone crotonylation, will improve our understanding of processes that regulate this modification. Although crotonyl-CoA is an intermediate in metabolic processes including fatty acid β-oxidation and degradation of the essential amino acids lysine or tryptophan, the contribution of these pathways in mediating histone crotonylation in mammalian cells remains unknown. If a link between metabolism and histone crotonylation is established then this modification is likely to have important implications in prevalent diseases associated with metabolic dysfunction, which include cancer and cardiovascular disease.

In summary, histone crotonylation has an active role in gene regulation that is functionally distinct from histone acetylation. The pronounced ability of histone Kcr to regulate gene expression, which outperforms histone Kac, has helped to establish this modification as an important regulator of cellular signalling and tissue function. However, further studies are needed to better define how histone crotonylation is regulated and its association with diverse physiological and pathological processes.

## Author Contributions

AN wrote the manuscript. JB made suggestions and edited the manuscript. Both authors read and approved the final manuscript.

## Conflict of Interest

The authors declare that the research was conducted in the absence of any commercial or financial relationships that could be construed as a potential conflict of interest.

## References

[B1] Abi Khalil C. (2014). The emerging role of epigenetics in cardiovascular disease. *Ther. Adv. Chronic Dis.* 5 178–187. 10.1177/2040622314529325 24982752 PMC4049125

[B2] Abu-Zhayia E. R. Machour F. E. Ayoub N. (2019). HDAC-dependent decrease in histone crotonylation during DNA damage. *J. Mol. Cell Biol.* 11 804–806. 10.1093/jmcb/mjz019 30864665 PMC6821229

[B3] Allfrey V. G. Faulkner R. Mirsky A. E. (1964). Acetylation and methylation of histones and their possible role in the regulation of rna synthesis. *Proc. Natl. Acad. Sci. U.S.Am.* 51 786–794. 10.1073/pnas.51.5.786 14172992 PMC300163

[B4] Andrews F. H. Shinsky S. A. Shanle E. K. Bridgers J. B. Gest A. I Tsun K. (2016). The Taf14 YEATS domain is a reader of histone crotonylation. *Nat. Chem. Biol.* 12 396–398. 10.1038/nchembio.2065 27089029 PMC4871749

[B5] Bannister A. J. Kouzarides T. (2011). Regulation of chromatin by histone modifications. *Cell Res.* 21 381–395. 10.1038/cr.2011.22 21321607 PMC3193420

[B6] Bao X. Liu Z. Zhang W. Gladysz K. Fung Y. M. E. Tian G. (2019). Glutarylation of histone H4 Lysine 91 regulates chromatin dynamics. *Mol. Cell* 76 660–675.e9. 10.1016/j.molcel.2019.08.018 31542297

[B7] Bao X. Wang Y. Li X. Li X. M. Liu Z. Yang T. (2014). Identification of ‘erasers’ for lysine crotonylated histone marks using a chemical proteomics approach. *ELife* 3:e02999. 10.7554/eLife.02999 25369635 PMC4358366

[B8] Barnes C. E. English D. M. Cowley S. M. (2019). Acetylation & co: an expanding repertoire of histone acylations regulates chromatin and transcription. *Essays Biochem.* 63 97–107. 10.1042/EBC20180061 30940741 PMC6484784

[B9] Caron C. Pivot-Pajot C. van Grunsven L. A. Col E. Lestrat C. Rousseaux S. (2003). Cdyl: a new transcriptional co-repressor. *EMBO Rep.* 4 877–882. 10.1038/sj.embor.embor917 12947414 PMC1326355

[B10] Chen Y. Sprung R. Tang Y. Ball H. Sangras B. Kim S. C. (2007). Lysine propionylation and butyrylation are novel post-translational modifications in histones. *Mol. Cell. Proteomics* 6 812–819. 10.1074/mcp.M700021-MCP200 17267393 PMC2911958

[B11] Curradi M. Izzo A. Badaracco G. Landsberger N. (2002). Molecular mechanisms of gene silencing mediated by DNA methylation. *Mol. Cell. Biol.* 22 3157–3173. 10.1128/mcb.22.9.3157-3173.2002 11940673 PMC133775

[B12] Dai L. Peng C. Montellier E. Lu Z. Chen Y. Ishii H. (2014). Lysine 2-hydroxyisobutyrylation is a widely distributed active histone mark. *Nat. Chem. Biol.* 10 365–370. 10.1038/nCHeMBIO.1497 24681537

[B13] De Ruijter A. J. M. Van Gennip A. H. Caron H. N. Kemp S. Van Kuilenburg A. B. P. (2003). Histone deacetylases (HDACs): characterization of the classical HDAC family. *Biochem. J*. 370 737–749. 12429021 10.1042/BJ20021321 PMC1223209

[B14] Edrissi B. Taghizadeh K. Dedon P. C. (2013). Quantitative analysis of histone modifications: formaldehyde is a source of pathological N6-formyllysine that is refractory to histone deacetylases. *PLoS Genet.* 9:e1003328. 10.1371/journal.pgen.1003328 23468656 PMC3585032

[B15] Feldman J. L. Baeza J. Denu J. M. (2013). Activation of the protein deacetylase SIRT6 by long-chain fatty acids and widespread deacylation by mammalian sirtuins. *J. Biol. Chem.* 288 31350–31356. 10.1074/jbc.C113.511261 24052263 PMC3829447

[B16] Fellows R. Denizot J. Stellato C. Cuomo A. Jain P. Stoyanova E. (2018). Microbiota derived short chain fatty acids promote histone crotonylation in the colon through histone deacetylases. *Nat. Commun.* 9:105. 10.1038/s41467-017-02651-5 29317660 PMC5760624

[B17] Flynn E. M. Huang O. W. Poy F. Oppikofer M. Bellon S. F. Tang Y. (2015). A subset of human bromodomains recognizes butyryllysine and crotonyllysine histone peptide modifications. *Structure* 23 1801–1814. 10.1016/j.str.2015.08.004 26365797

[B18] Fu H. Tian C. L. Ye X. Sheng X. Wang H. Liu Y. (2018). Dynamics of telomere rejuvenation during chemical induction to pluripotent stem cells. *Stem Cell Rep* 11 70–87. 10.1016/j.stemcr.2018.05.003 29861168 PMC6066961

[B19] Goudarzi A. Zhang D. Huang H. Barral S. Kwon O. K. Qi S. (2016). Dynamic Competing Histone H4 K5K8 Acetylation and Butyrylation Are Hallmarks of Highly Active Gene Promoters. *Mol. Cell* 62 169–180. 10.1016/j.molcel.2016.03.014 27105113 PMC4850424

[B20] Gowans G. J. Bridgers J. B. Zhang J. Dronamraju R. Burnetti A. King D. A. (2019). Recognition of histone crotonylation by Taf14 links metabolic state to gene expression. *Mol. Cell* 76 909–921.e3. 10.1016/j.molcel.2019.09.029 31676231 PMC6931132

[B21] Huang H. Zhang D. Wang Y. Perez-Neut M. Han Z. Zheng Y. G. (2018). Lysine benzoylation is a histone mark regulated by SIRT2. *Nat. Commun.* 9:3374. 10.1038/s41467-018-05567-w 30154464 PMC6113264

[B22] Hunsberger J. G. Newton S. S. Bennett A. H. Duman C. H. Russell D. S. Salton S. R. (2007). Antidepressant actions of the exercise-regulated gene VGF. *Nat. Med.* 13 1476–1482. 10.1038/nm1669 18059283

[B23] Jackson S. P. Bartek J. (2009). The DNA-damage response in human biology and disease. *Nature* 461 1071–1078. 10.1038/nature08467 19847258 PMC2906700

[B24] Jiang G. Nguyen D. Archin N. M. Yukl S. A. éndez-Lagares G. M. Tang Y. (2018). HIV latency is reversed by ACSS2-driven histone crotonylation. *J. Clin. Invest.* 461 1071–1078. 10.1172/JCI98071 29457784 PMC5824862

[B25] Jiang H. Chen S. Lu N. Yue Y. Yin Y. Zhang Y. (2017). Reduced serum VGF levels were reversed by antidepressant treatment in depressed patients. *World J. Biol. Psychiatry* 18 586–591. 10.1080/15622975.2016.1224923 28635540

[B26] Jiang T. Zhou X. Taghizadeh K. Dong M. Dedon P. C. (2007). N-formylation of lysine in histone proteins as a secondary modification arising from oxidative DNA damage. *Proc. Natl. Acad. Sci. U.S.Am.* 104 60–65. 10.1073/pnas.0606775103 17190813 PMC1765477

[B27] Kaczmarska Z. Ortega E. Goudarzi A. Huang H. Kim S. Márquez J. A. (2017). Structure of P300 in complex with Acyl-CoA variants. *Nat. Chem. Biol.* 13 21–29. 10.1038/nchembio.2217 27820805 PMC5757799

[B28] Kebede A. F. Niebora A. Shahidian L. Z. Gras S. L. Richter F. Gómez D. A. (2017). Histone propionylation is a mark of active chromatin. *Nat. Struct. Mol. Biol.* 24 1048–1056. 10.1038/nsmb.3490 29058708

[B29] Kelly R. D. W. Chandru A. Watson P. J. Song Y. Blades M. Robertson N. S. (2018). Histone deacetylase (HDAC) 1 and 2 complexes regulate both histone acetylation and crotonylation in vivo. *Sci. Rep.* 8:14690. 10.1038/s41598-018-32927-9 30279482 PMC6168483

[B30] Klein B. J. Jang S. M. Lachance C. Mi W. Lyu J. Sakuraba S. (2019). Histone H3K23-specific acetylation by morf is coupled to H3K14 acylation. *Nat. Commun.* 10:4724. 10.1038/s41467-019-12551-5 31624313 PMC6797804

[B31] Kollenstart L. de Groot A. J. L. Janssen G. M. C. Cheng X. Vreeken K. Martino F. (2019). Gcn5 and Esa1 function as histone crotonyltransferases to regulate crotonylation-dependent transcription. *J. Biol. Chem.* 294 20122–20134. 10.1074/jbc.RA119.010302 31699900 PMC6937567

[B32] Li X. Li X. M. Jiang Y. Liu Z. Cui Y. Fung K. Y. (2018). Structure-guided development of YEATS domain inhibitors by targeting π-π-π stacking. *Nat. Chem. Biol.* 14 1140–1149. 10.1038/s41589-018-0144-y 30374167 PMC6503841

[B33] Li Y. Sabari B. R. Panchenko T. Wen H. Zhao D. Guan H. (2016). Molecular coupling of histone crotonylation and active transcription by AF9 YEATS domain. *Mol. Cell* 62 181–193. 10.1016/j.molcel.2016.03.028 27105114 PMC4841940

[B34] Li Y. Zhao D. Chen Z. Li H. (2017). YEATS domain: linking histone crotonylation to gene regulation. *Transcription* 8 9–14. 10.1080/21541264.2016.1239602 27661789 PMC5402991

[B35] Liu S. Yu H. Liu Y. Liu X. Zhang Y. Bu C. (2017). Chromodomain protein CDYL acts as a crotonyl-CoA hydratase to regulate histone crotonylation and spermatogenesis. *Mol. Cell* 67 853–866.e5. 10.1016/j.molcel.2017.07.011 28803779

[B36] Liu X. Wei W. Liu Y. Yang X. Wu J. Zhang Y. (2017). MOF as an evolutionarily conserved histone crotonyltransferase and transcriptional activation by histone acetyltransferase-deficient and crotonyltransferase-competent CBP/P300. *Cell Discov.* 3:17016. 10.1038/celldisc.2017.16 28580166 PMC5441097

[B37] Liu Y. Li M. Fan M. Song Y. Yu H. Zhi X. (2019). Chromodomain Y-like protein–mediated histone crotonylation regulates stress-induced depressive behaviors. *Biol. Psychiatr.* 85 635–649. 10.1016/j.biopsych.2018.11.025 30665597

[B38] Liu Y. Liu S. Yuan S. Yu H. Zhang Y. Yang X. (2017). Chromodomain protein CDYL is required for transmission/restoration of repressive histone marks. *J. Mol. Cell Biol.* 9 178–194. 10.1093/JMCB/MJX013 28402439

[B39] Machour F. E. Ayoub N. (2020). Transcriptional regulation at DSBs: mechanisms and consequences. *Trends Genet*. 36 981–997. 10.1016/j.tig.2020.01.001 32001024

[B40] Madsen A. S. Olsen C. A. (2012). Profiling of substrates for zinc-dependent lysine deacylase enzymes: HDAC3 exhibits decrotonylase activity in vitro. *Angew. Chem. Int. Ed. Engl.* 51 9083–9087. 10.1002/anie.201203754 22890609

[B41] Montellier E. Boussouar F. Rousseaux S. Zhang K. Buchou T. Fenaille F. (2013). Chromatin-to-nucleoprotamine transition is controlled by the histone H2B Variant TH2B. *Genes Dev.* 27 1680–1692. 10.1101/gad.220095.113 23884607 PMC3744726

[B42] Montellier E. Rousseaux S. Zhao Y. Khochbin S. (2012). Histone crotonylation specifically marks the haploid male germ cell gene expression program: post-meiotic male-specific gene expression. *Bioessays* 34 187–193. 10.1002/bies.201100141 22170506

[B43] Mulligan P. Westbrook T. F. Ottinger M. Pavlova N. Macia E. Shi Y. J. (2019). CDYL bridges REST and histone methyltransferases for gene repression and suppression of cellular transformation. *Mol. Cell* 32 718–726. 10.1016/j.molcel.2008.10.025.CDYL PMC6595072 19061646

[B44] Roth S. Y. Denu J. M. Allis C. D. (2001). *Histone A**cetyltransferases.* Available online at: www.annualreviews.org (accessed October 5, 2020).

[B45] Ruiz-Andres O. Sanchez-Niño M. D. Cannata-Ortiz P. Ruiz-Ortega M. Egido J. Ortiz A. (2016). Histone lysine crotonylation during acute kidney injury in mice. *Dis. Model. Mech.* 9 633–645. 10.1242/dmm.024455 27125278 PMC4920150

[B46] Sabari B. R. Tang Z. Huang H. Yong-Gonzalez V. Molina H. Kong H. E. (2015). Intracellular crotonyl-CoA stimulates transcription through P300-catalyzed histone crotonylation. *Mol. Cell* 58 203–215. 10.1016/j.molcel.2015.02.029 25818647 PMC4501262

[B47] Sabari B. R. Zhang D. Allis C. D. Zhao Y. (2017). Metabolic regulation of gene expression through histone acylations. *Nat. Rev. Mol. Cell Biol*. 18 90–101. 10.1038/nrm.2016.140 27924077 PMC5320945

[B48] Simithy J. Sidoli S. Yuan Z. F. Coradin M. Bhanu N. V. Marchione D. M. (2017). Characterization of histone acylations links chromatin modifications with metabolism. *Nat. Commun.* 8:1141. 10.1038/s41467-017-01384-9 29070843 PMC5656686

[B49] Sutendra G. Kinnaird A. Dromparis P. Paulin R. Stenson T. H. Haromy A. (2014). A nuclear pyruvate dehydrogenase complex is important for the generation of acetyl-CoA and Histone acetylation. *Cell* 158 84–97. 10.1016/j.cell.2014.04.046 24995980

[B50] Suzuki Y. Horikoshi N. Kato D. Kurumizaka H. (2016). Crystal structure of the nucleosome containing histone H3 with crotonylated lysine 122. *Biochem. Biophys. Res. Communications* 469 483–489. 10.1016/j.bbrc.2015.12.041 26694698

[B51] Tan J. McKenzie C. Potamitis M. Thorburn A. N. Mackay C. R. Macia L. (2014). The role of short-chain fatty acids in health and disease. *Adv. Immunol.* 121 91–119. 10.1016/B978-0-12-800100-4.00003-9 24388214

[B52] Tan M. Luo H. Lee S. Jin F. Yang J. S. Montellier E. (2011). Identification of 67 histone marks and histone lysine crotonylation as a new type of histone modification. *Cell* 146 1016–1028. 10.1016/j.cell.2011.08.008 21925322 PMC3176443

[B53] Tan M. Peng C. Anderson K. A. Chhoy P. Xie Z. Dai L. (2014). Lysine glutarylation is a protein posttranslational modification regulated by SIRT5. *Cell Metab.* 19 605–617. 10.1016/j.cmet.2014.03.014 24703693 PMC4108075

[B54] Turner A. W. Margolis D. M. (2017). Chromatin regulation and the histone code in HIV latency. *Yale J. Biol. Med*. 90 229–243. 28656010 PMC5482300

[B55] Turner J. M. A. (2007). Meiotic sex chromosome inactivation. *Development* 134 1823–1831. 10.1242/dev.000018 17329371

[B56] Wei W. Liu X. Chen J. Gao S. Lu L. Zhang H. (2017). Class i histone deacetylases are major histone decrotonylases: evidence for critical and broad function of histone crotonylation in transcription. *Cell Res.* 27 898–915. 10.1038/cr.2017.68 28497810 PMC5518989

[B57] Wellen K. E. Hatzivassiliou G. Sachdeva U. M. Bui T. V. Cross J. R. Thompson C. B. (2009). ATP-citrate lyase links cellular metabolism to histone acetylation. *Science* 324 1076–1080. 10.1126/science.1164097 19461003 PMC2746744

[B58] Xie Z. Dai J. Dai L. Tan M. Cheng Z. Wu Y. (2012). Lysine succinylation and lysine malonylation in histones. *Mol. Cell. Proteomics* 11 100–107. 10.1074/mcp.M111.015875 22389435 PMC3418837

[B59] Xie Z. Zhang D. Chung D. Tang Z. Huang H. Dai L. (2016). Metabolic regulation of gene expression by histone lysine β-hydroxybutyrylation. *Mol. Cell* 62 194–206. 10.1016/j.molcel.2016.03.036 27105115 PMC5540445

[B60] Xiong X. Panchenko T. Yang S. Zhao S. Yan P. Zhang W. (2016). Selective recognition of histone crotonylation by double PHD fingers of MOZ and DPF2. *Nat. Chem. Biol.* 12 1111–1118. 10.1038/nchembio.2218 27775714 PMC5253430

[B61] Zhang Q. Zeng L. Zhao C. Ju Y. Konuma T. Zhou M. M. (2016). Structural insights into histone crotonyl-lysine recognition by the AF9 YEATS domain. *Structure* 24 1606–1612. 10.1016/j.str.2016.05.023 27545619 PMC5014688

[B62] Zhang Y. Yang X. Gui B. Xie G. Zhang D. Shang Y. (2011). Corepressor protein CDYL functions as a molecular bridge between polycomb repressor complex 2 and repressive chromatin mark trimethylated histone lysine 27. *J. Biol. Chem.* 286 42414–42425. 10.1074/jbc.M111.271064 22009739 PMC3234934

[B63] Zhao D. Guan H. Zhao S. Mi W. Wen H. Li Y. (2016). YEATS2 is a selective histone crotonylation reader. *Cell Res.* 26 629–632. 10.1038/cr.2016.49 27103431 PMC4856769

[B64] Zhao D. Li Y. Xiong X. Chen Z. Li H. (2017). YEATS domain—A histone acylation reader in health and disease. *J. Mol. Biol*. 429 1994–2002. 10.1016/j.jmb.2017.03.010 28300602

[B65] Zhao S. Zhang X. Li H. (2018). Beyond histone acetylation—writing and erasing histone acylations. *Curr. Opin. Struct. Biol.* 53 169–177. 10.1016/j.sbi.2018.10.001 30391813

